# Reduced transcriptional activity in the p53 pathway of senescent cells revealed by the MDM2 antagonist nutlin-3

**DOI:** 10.18632/aging.100091

**Published:** 2009-09-25

**Authors:** Baoying Huang, Lyubomir T. Vassilev

**Affiliations:** Discovery Oncology, Roche Research Center, Hoffmann-La Roche Inc., Nutley, NJ 07110, USA

**Keywords:** p53, MDM2, senescence, apoptosis, cancer

## Abstract

The p53 tumor suppressor plays a key role in induction and maintenance of cellular senescence but p53-regulated response to stress in senescent cells is poorly understood.  Here, we use the small-molecule MDM2 antagonist, nutlin-3a, to selectively activate p53 and probe functionality of the p53 pathway in senescent human fibroblasts, WI-38.  Our experiments revealed overall reduction in nutlin-induced transcriptional activity of nine p53 target genes and four p53-regulated microRNAs, indicating that not only p53 protein levels but also its ability to activate transcription are altered during senescence.  Addition of nutlin restored doxorubicin-induced p53 protein and transcriptional activity in senescent cells to the levels in early passage cells but only partially restored its apoptotic activity, suggesting that changes in both upstream and downstream p53 signaling during senescence are responsible for attenuated response to genotoxic stress.

## Introduction

Cellular senescence is a state in which cells irreversibly lose their ability to proliferate after a finite number of divisions in culture [1]. Senescent cells are viable and metabolically active, but unable to replicate their DNA [2,3]. They are distinguished from their proliferating counterparts by increased size, flat morphology, elevated activity of senescence-associated β-galactosidase (SA-β-Gal) [4], and formation of characteristic senescence-associated heterochromatin foci (SAHF) [5].   Telomere shortening, a consequence of repeated cycles of DNA replication is thought to be a critical trigger of senescence [6,7] which also involves activation of two major tumor suppressor pathways, p53 and Rb [2,8,9]. Cellular senescence may lead to aging, a process associated with a reduced capacity of tissue regeneration and decline of physiological functions [9]. Although a direct link between senescence and aging has not been established, it has been suggested that senescence contributes to aging in several ways [10]. Accumulation of senescence cells may change tissue morphology and reduce its functionality. Senescence may also compromise tissue repair and renewal due to the lack of cell division.  Markers of senescence such as increased SA-β-Gal staining have been frequently observed in aging tissues [4]. Therefore, senescence has been considered a cellular counterpart of aging, and represents a model system to study the molecular events leading to aging [9].

The tumor suppressor p53 is a key mediator of cellular senescence.  It is in the center of a complex signal transduction network, the p53 pathway, which controls cellular response to stress by inducing cell cycle arrest, apoptosis or senescence [11,12].  p53 is a potent transcription factor regulating the expression of multiple target genes in response to diverse stresses.  Recently, it has been reported that p53 can activate the transcription of microRNA genes (e.g. miR-34 family), with possible roles in apoptosis and/or cellular senescence [13,14]. p53 activation is a critical step in induction of cellular senescence because its inactivation allows cells to bypass senescence [15].  Knockdown of p53 reverses established senescence, indicating that p53 activity is also required for maintenance of the senescence state [16].  However, despite the need for active p53 and its well established pro-apoptotic function, senescent cells appear resistant to p53-dependent apoptosis induced by various stresses including DNA damage [17-19]. These observations have raised the question: Is p53 apoptotic function compromised in senescent cells?  One possible way to disable p53 apoptotic activity is by defective upstream p53 signaling. Indeed, previous studies have suggested that resistance to apoptosis may be due to inability to stabilize p53 in senescent cells in response to DNA damaging agents [17].  Similarly, significant decrease in p53-dependent apoptosis in response to ionizing radiation has been seen in aging compared to young mouse tissues [20]. Expression levels of p53 target genes (e.g. p21, MDM2, Cyclin G1) have been reduced upon radiation treatment concomitant with lower ATM activity in older mouse tissues. It is also possible that p53 transcriptional activity itself is decreased in aging tissues.  It has been reported that p53 phosphorylation status in senescence differs from that of proliferating cells [21].  Another possibility for resistance to apoptosis could be the heterochromatinization and gene silencing in senescence cells of aging tissues that may prevent transcription of some p53 target genes despite the presence of activated p53. To distinguish between these possibilities one need to separate upstream from downstream signaling events in the p53 pathway.  The MDM2 antagonist, Nutlin-3a, which stabilizes p53 by preventing its MDM2-dependent degradation, offers such a tool [22].

Nutlin is a small-molecule inhibitor of the p53-MDM2 interaction that protects the tumor suppressor from its negative regulator, MDM2, stabilizes p53 and activates the p53 pathway [23,24]. Nutlin is not genotoxic and does not cause p53 phosphorylation [25] but effectively activates the two major p53 functions: cell cycle arrest and apoptosis [26].  It upregulates p53 without the need for upstream signaling events, and allows to investigate the functionality of downstream p53 signaling in senescent cells.  Here, we use human lung fibroblasts, WI-38, as a model system to study p53 transcriptional activity and apoptosis in senescence.  We find that p53 is functional as a transcription factor in senescent cells, but its ability to induce many target genes and apoptosis is attenuated.

## Results

### Expression of p53 target genes in senescent WI-38 cells

**Figure 1.**
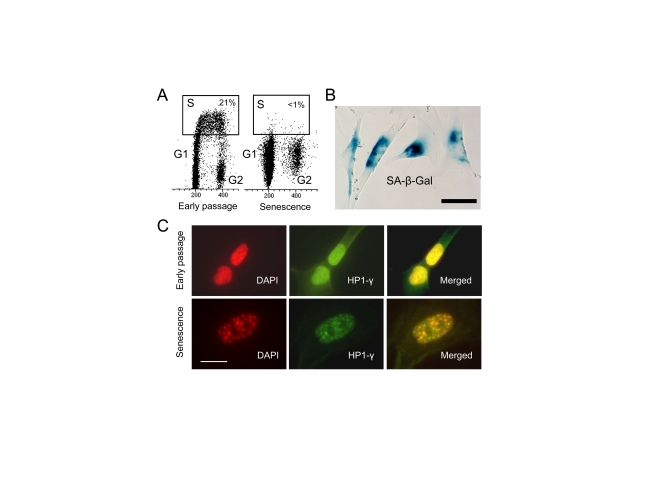
** WI-38 cells cease to proliferate after extensive in vitro passaging and become senescent. **(**A**) Cell cycle analysis after BrdU incorporation in early passage and senescent cells.  S phase cells are within the rectangle. (**B**) Senescent WI-38 cells stain for SA-β-Gal activity. Scale bar is 50 μm.  (**C**) SAHF in senescent WI-38 cells. Early passage and senescent cells were immunostained with anti-HP-1γ antibody (green) and counter stained with DAPI (red). Scale bar is 20  μm.

As a model system to study p53 function in senescence, we have chosen human embryonal lung fibroblast, WI-38 [27]. They were subjected to extensive serial passages in culture for a period of 3 months.  The first signs of senescence, slow growth, enlarged size and flat morphology, were noted after 45 to 60 days.  SA-β-Gal staining was then used to monitor the state of the population.  After an additional month of passaging, cells apparently ceased to proliferate and showed a typical senescence phenotype (data not shown). Bromodeoxyuridine (BrdU) labeling revealed that less than 1% of the cell population is in S phase, indicating that they have exited the cell cycle (Figure 1A).  All cells stained intensely for SA-β-Gal activity (Figure 1B).  To assure that the cells have acquired true replicative senescence we analyzed them for the presence of senescence-associated heterochromatin foci (SAHF) considered one of the most reliable markers of senescence [5].  Presence of SAHF indicates that irreversible changes in chromatin organization and gene function have taken place [5].  These foci contain several heterochromatin markers such as hypoacetylated histones, H3 methylation, and heterochromatin protein 1 (HP1).  It has been shown that several E2F target genes are embedded into these heterochromatin structures thus prohibiting E2F from binding to gene promoters [5].  Furthermore, DNA from senescent cells has been found more resistant to digestion by micrococcal nuclease, suggesting less accessibility of DNA [5].  Immunostaining of WI-38 cells revealed multiple SAHF foci in which HP1-γ and DAPI signals overlapped, suggesting that heterochromatinization has been completed (Figure 1C).  The typical senescence morphology, intense SA-β-Gal staining, lack of DNA replication and SAHF, all indicated that the population of WI-38 cells is in a state of replicative senescence.

**Figure 2.**
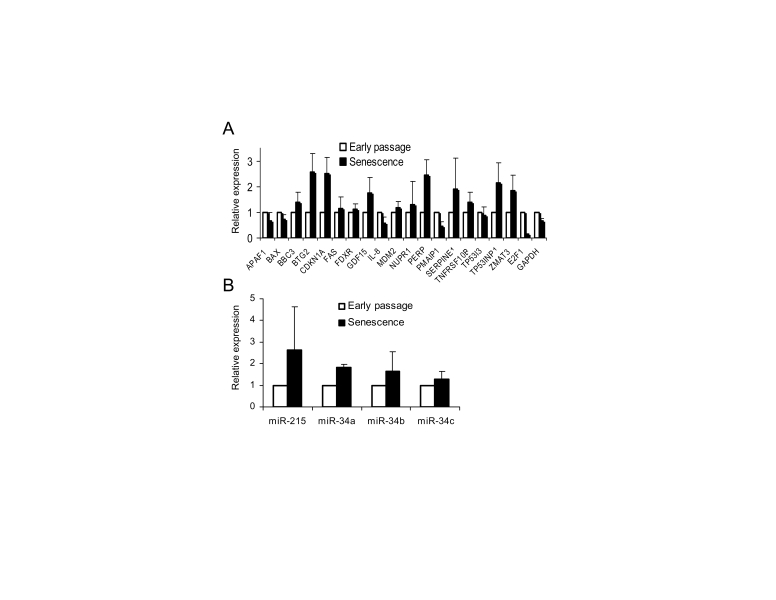
** Transcriptional activity of p53 target genes in senescent WI-38 fibroblasts**. (**A**) Basal transcription of p53 target genes is not compromised in senescent cells.  Total RNA from early passage and senescent WI-38 cells was isolated and the expression of specific mRNAs was determined by quantitative PCR. Expression levels of each individual mRNA (from early passage and senescent cells) were normalized to 18S rRNA.  Expression levels in senescent cells were calculated as fold change from the expression levels in early passage cells.  The standard deviation (SD) was calculated from four independent experiments.  (**B**) Basal expression levels of p53-regulated microRNA in senescent cells. Expression levels of individual microRNAs from early passage and senescent cells were determined by quantitative PCR, and normalized to RNU48 as an internal control.  Expression levels in senescent cells were calculated and presented as in (**A**).

We then examined the expression levels of 18 known p53 target genes in early passage and senescent WI-38 cells using quantitative real-time PCR (Figure 2). The list included genes involved in p53 regulation (MDM2), cell cycle arrest (BTG2, CDKN1A/p21), apoptosis (BAX, BBC3/Puma, FAS), and others.  Of the 18 genes tested, 14 showed similar or higher expression level in senescent cells compared to early passage cells (Figure 2A). Three genes, APAF1, BAX and IL-8, had slightly reduced expression levels in senescent cells (60%-70% of early passage), while only one gene, PMAIP1 (NOXA), showed more than two-fold lower expression. The transcription of cell cycle genes, p21 and BTG2, was elevated more than two-fold in senescent cells, consistent with a previous report that p53 is preferentially recruited to promoter of growth arrest genes during replicative senescence [28].  As a control, the expression level of a housekeeping gene, GAPDH, was also determined.  It showed slightly lower expression in senescent (65%) compared to early passage cells. The transcription level of E2F1, usually repressed in senescent cells [5], was found reduced by approximately 90% compared to early passage cell.  We also examined basal expression levels of the p53 target microRNA genes: miR-34a, b, c [13,29], and miR-215 [30,31]], previously reported to contribute to cell cycle arrest and/or apoptotic activity of p53.  All four microRNAs were expressed at similar (miR-34c) or higher (miR215, miR34a and b) levels in senescent cells compared to early passage cells (Figure 2B). These results indicated that despite SAHF formation, the basal level of transcription for the tested p53-regulated genes was equal or higher in senescent cells than their early passage counterparts.

### Decline in transcriptional response to nutlin-induced p53 in senescent cells

It has been well documented that tumor suppressor function of p53 depends on its ability to activate the transcription of multiple target genes involved in cell cycle arrest and apoptosis in response to diverse forms of oncogenic stress [32].  A recent study has indicated compromised p53 function in aging mouse tissues [20].  This could result from changes in the upstream and/or downstream p53 signaling, leading to inadequate p53 accumulation, inactive p53 protein, problems with regulation of transcription, or combination of the above.  Here, we use senescent cells under well controlled condition and the non-genotoxic p53 activator nutlin-3a to address these possibilities. Nutlin selectively inhibits the MDM2-p53 interaction and directly stabilizes p53 by preventing its degradation regardless of p53 upstream signaling. Therefore, nutlin allows examining the functionality of downstream p53 signaling.

**Figure 3.**
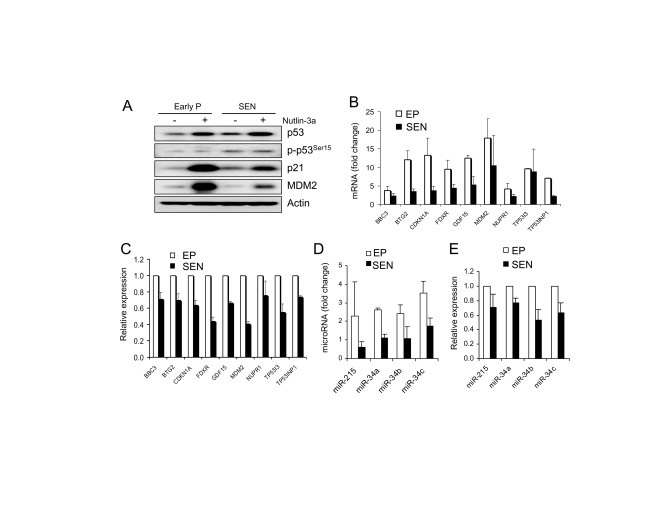
Transcriptional activity of nutlin-induced p53 is attenuated in senescent WI-38 cells. (**A**) Protein level of p53 and two target genes, p21 and MDM2, in early passage and senescent cells. Cells were incubated in the presence of 10 μM nutlin-3a for 24 hours, lysed and subjected to Western blotting as described. (**B**) Induction of p53 target genes by nutlin-3a is decreased in senescent cells. Cells were treated as in (**A**), RNA was extracted and subjected to quantitative PCR.  Fold induction is calculated as change in post compared to pre-treatment expression levels, both normalized to 18S rRNA. (**C**) Total expression levels of p53 target genes are reduced in nutlin-3a treated senescent cells. Cells were treated as in (**B**).  Gene expression levels after exposure to nutlin are shown normalized to expression levels in early passage cells (100%).  (**D**) Induction of p53 regulated microRNAs by nutlin-3a is decreased in senescent cells. Cells were treated as in (**B**).  MicroRNA expression is determined by quantitative PCR and normalized to RNU48. Fold induction is calculated as in (**B**).(**E**) Total expression levels of p53-regulated microRNA are reduced in nutlin-treated senescent cells.  Cells are treated as in (**B**), microRNA determined as in (**D**), and data presented as in (**C**).

Treatment of early passage and senescent WI-38 cells with 10 μM nutlin-3a for 24 hours elevated p53 protein in both senescent and early passage cells (Figure 3A). Induced p53 protein levels were comparable in both cell types, indicating that p53 can be stabilized in senescent cells in a similar way as in early passage cells.  By generating practically equal amounts of p53 protein, nutlin allowed for examining downstream transcriptional activation events. We have shown previously that nutlin-3a does not induce phosphorylation of p53 on six key N-terminal residues in proliferating cancer cells but retains its ability to activate cell cycle arrest and apoptosis [25].  Here again, we see no change in the level of phospho-p53Ser15 after nutlin treatment.  However, there is a slight upregulation of p53Ser15 in senescent cells likely due to stress during continuous cell passage.

The change in the transcription levels of 18 p53 target genes (Figure 2) were determined in early passage WI-38 cells after 24 hours of exposure to nutlin-3a by quantitative PCR. Nutlin induced 9 out of 18 genes, BBC3 (PUMA), BTG2, CDKN2A (p21), FDXR, GDF-15, MDM2, NUPR1, TP53I3 and TP53INP1, greater than 2-fold (data not shown).  These genes were selected for further analysis in the senescent cells (Figure 3B).  Comparison of nutlin-induced expression levels revealed that 8/9 genes had reduced induction in senescent compared to early passage cells (Figure 3B).  BTG2 and CDKN2A (p21) were induced approximately 12 and 13-fold, respectively in early passage cells, but only 3.5 and 3.8-fold in the senescent cells.  However, some of these genes have shown higher basal expression in senescent than early passage cells, suggesting that they may have the same or even higher overall expression level in senescent cells despite the reduced induction. Therefore, we compared the normalized expression levels of these genes rather than fold change. This analysis showed that the expression levels of the 8 genes in nutlin-treated senescent cells range from 41% (MDM2) to 71% (BBC3) of the expression levels in early passage cells (Figure 3C).  This is in agreement with the reduced protein levels of p21 and MDM2 in nutlin-treated senescent cells (Figure 3A).  These results suggest overall reduction of transcription activity of p53 target genes in the senescent cells.

We also evaluated nutlin-induced expression of four microRNA genes (miR-34a, b, c and miR-215) before and after treatment in both early passage and senescent cells.  All tested microRNAs were induced more than 2-fold in early passage cells but not in the senescent cells (Figure 3D).  Normalized expression levels of these microRNAs in senescent cells ranged from 53% (miR-34b) to 77% (miR-34a) of the expression levels in early passage cells (Figure 3E), consistent with a decrease in p53-induced transcriptional activity in senescent cells. Therefore, despite the comparable levels of nutlin-induced p53 protein between early passage and senescent cells, p53's ability to transactivate its target genes was attenuated in senescent cells.

### Decline in apoptotic response of senescent cells to DNA damage correlates with ineffective p53 stabilization

**Figure 4.**
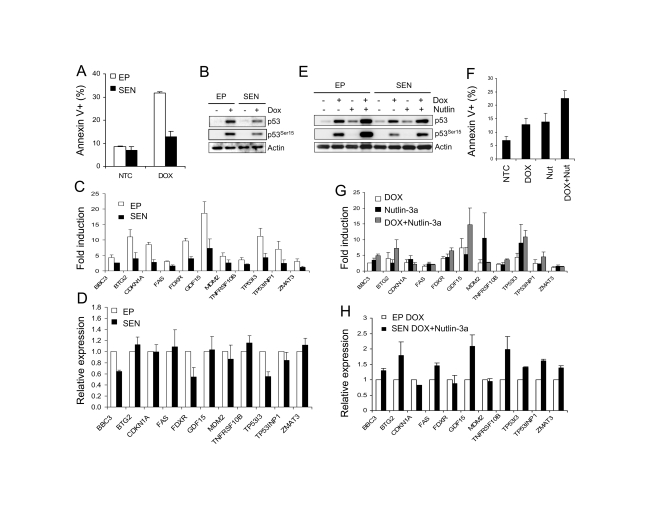
Doxorubicin-induced apoptosis in senescent cells. (**A**) Senescent WI-38 cells are resistant to doxorubicin-induced apoptosis. Early passage and senescent cells were incubated in the presence of 300 nM doxorubicin for 72 hours and the fraction of apoptotic cells was determined by the Annexin V assay.  (**B**)  Western blot analysis of early passage and senescent WI-38 cells treated with 300 nM doxorubicin for 24 hours.  (**C**) Transcriptional activity of p53 target genes in doxorubicin-treated senescent WI-38 cells. Early passage and senescent cells were exposed to 300 nM doxorubicin, RNA was extracted for and data analyzed as in Figure 3B.  (**D**) Effect of doxorubicin on the relative expression levels of p53 target genes in senescent cells. Cells were treated as in (**C**) and data calculated and presented as in Figure 3C.  (**E**) Nutlin raises doxorubicin-induced p53 protein level in senescent cells.  Early passage and senescent WI-38 cells were exposed to 300 nM doxorubicin, 10 μM Nutlin-3a, or combination of both for 24 hours prior to collection for Western analysis. (**F**) Nutlin increases apoptosis induced by doxorubicin in senescent cells. Senescent WI-38 cells were treated with 10 μM nutlin-3a, 300 nM doxorubicin or 10 μM nutlin-3a plus 300 nM doxorubicin for 72 hours and the apoptotic cell fractions were measured by the Annexin-V assay.  (**G**) Nutlin restores transcriptional response to doxorubicin-induced p53 in senescent cells. Senescent cells were treated with 10 μM nutlin-3a, 300 nM doxorubicin or 10 μM nutlin-3a plus 300 nM doxorubicin for 24 hours and expression levels of indicated genes were determined by quantitative PCR, normalized, and calculated as fold change.  (**H**) Nutlin restores the transcription of doxorubicin-induced p53 target genes in senescent cells to early passage levels. Early passage cells were exposed to 300 nM doxorubicin and senescent cells to 300 nM doxorubicin plus 10 μM nutlin-3a.  24 hours after treatment, mRNA levels were determined by quantitative PCR and normalized to expression levels in early passage cells (100%).

It has been shown that senescent cells are more resistant to p53-dependent apoptosis induced by UV, H_2_O_2_, and genotoxic drugs [17,18] but the molecular mechanisms behind this resistance are not fully understood. We asked if the decline in transcriptional response to p53 activation contributes to the resistance to apoptosis induced by DNA damage.  To this end, we examined p53-dependent transcription and apoptosis in senescent WI-38 cells in response to the genotoxic drug doxorubicin.  After 72 hours of exposure to high dose of doxorubicin (300 nM), the early passage WI-38 cells showed approximately 30% apoptotic (Annexin V-positive) cells (Figure 4A). The apoptotic fraction dropped to approximately 15% in the senescent cells, only a slight increase over the basal control level.  Western blot analysis revealed lower levels of p53 protein and its Ser-15 phosphorylated form (Figure 4B), suggesting inefficient upstream p53 signaling as a possible cause of reduced apoptotic response.  We then examined mRNA levels of the 18 p53 target genes, and found that 11 genes were induced greater than 2-fold in early passage cells (Figure 4C).  Gene activation was sig-  nificantly decreased in senescent cells where only 7 genes were induced more than 2-fold (Figure 4C). Upon examination of the normalized expression levels of these genes, we found that cell cycle arrest genes BTG2 and p21 are similar in senescent and early passage cells (Figure 4D).  However, overall expression levels of apoptosis-related genes (BBC3, TP53I3 and FDXR) were reduced approximately 50% in senescent cells (Figure 4D). Thus the decrease in apoptotic activity of doxorubicin in senescent cells correlated with a decline in transcription of p53 target genes associated with apoptosis.

To examine if reduced apoptosis is due to reduction in activated p53 protein, its transcriptional activity, or changes in other components of downstream apoptotic signaling, we tested a combination of doxorubicin and nutlin.   By inhibiting p53-MDM2 binding, nutlin can effectively stabilize p53 even in case of malfunctioning upstream p53 signaling.  Therefore, nutlin could restore possible defects in upstream signaling and raise the level of doxorubicin-induced p53.  Indeed, nutlin-doxorubicin combination induced higher p53 protein level in both early passage and senescent cells (Figure 4E).  Although nutlin did not induce Ser-15 phosphorylation, it stabilized the phosphorylated p53 induced by doxorubicin in both early passage and senescent cells (Figure 4E). Thus nutlin/doxorubicin combination generated p53 protein levels comparable to doxorubicin-induced p53 in early passage cells and should restore both transcriptional and the apoptotic response if they are reduced due to lower p53 protein levels.  In agreement with this expectation, the apoptotic fraction in senescent cells subjected to doxorubicin-nutlin combination increased to nearly 25% (Figure 4F), approaching the levels in early passage cells treated with doxorubicin alone (Figure 4A). Induced mRNA levels of the majority p53 target genes were found higher in the senescent cells exposed to doxorubicin-nutlin combination compared to doxorubicin alone, indicating higher transcriptional activity of the elevated p53 (Figure 4G).  When gene transcription was normal-ized, all 11 genes showed the same or higher levels compared with early passage cells (Figure 3H), suggesting that reduced levels of activated p53 protein are the likely cause of reduced transcriptional activity in senescent cells.  The restoration of p53 protein level and transcriptional response in senescent cells by nutlin/doxorubicin combination correlated with partial restoration of doxorubicin apoptotic activity in the senescent cell population.  This indicated that attenuated p53 stabilization is a major contributor to the reduced apoptotic response to doxorubicin in senescent WI-38 fibroblasts. However, the incomplete restoration of apoptosis suggests that factors other than p53 protein level and transcriptional activity might also contribute to the overall level of apoptosis.

## Discussion

The p53 tumor suppressor and the pathway it controls play a critical role in protection from cancer development by induction of cell cycle arrest, apoptosis or senescence in response to diverse oncogenic stresses [11,12].  Activation of the p53 pathway is essential for both induction and maintenance of senescence [3,16].  However, the functionality of the pathway e.g. ability to respond to stress and induce apoptosis in senescent cell is not well understood.   Experiments with senescent WI-38 fibroblasts have revealed that their ability to respond to genotoxic stresses by induction of apoptosis is compromised most likely due to inefficient p53 stabilization [17].  Recently, Feng et al. [20] have made similar observations by comparing p53 response to γ and UV radiation in young and aging mouse cells and tissues.  They concluded that inefficient p53 stabilization due to decreased ATM activity is the likely cause for declining apoptotic activity.  These studies have used radiation or genotoxic drugs known to have multiple mechanisms and do not allow to distinguish between defects in upstream or downstream p53 signaling.  Here, we use the non-genotoxic MDM2 antagonists, nutlin-3a, to investigate p53 functionality in senescent WI-38 fibroblasts.  By blocking the physical interaction between p53 and MDM2, nutlin stabilizes p53 independently of any upstream signaling events thus allowing to probe the functionality of the pathway downstream of p53.  Because of its high target selectivity, nutlin represents an excellent tool for studying p53 regulation and function in diverse cellular context under well control conditions [22].

We generated senescent WI-38 fibroblasts by continuous passages in vitro until the cells exited cycling and acquired a clear senescence phenotype confirmed by expression of senescence markers and SAHF (Figure 1).  We then looked at the basal level of expression of 18 p53 target genes in the senescent state.  One can speculate that heterochromatin structure in senescent cells may reduce the accessibility to promoters of p53 target genes and thus compromise the p53 transcription activity.  Our comparison of basal expression levels between early passage and senescent cells showed that the majority of the examined 18 p53 target genes are expressed at similar or higher levels in senescent cells (Figure 2).  Actually, several genes were expressed at higher basal level in senescent cells (e.g. p21, BTG2, PERP).  This may be partially due to the slightly higher p53 protein levels (Figure 3A) or higher affinity to promoters of some cell cycle related genes in senescence [28]. Similarly, we found that the basal expression levels of p53 regulated microRNAs are higher in senescent cells (Figure 2B). These results suggest that despite the appearance of multiple SAHF reflecting changes in chromatin structure the basal level of majority p53 target genes is not repressed.

To evaluate the transcriptional activity of p53 without the effect of altered upstream signaling, we compared p53-induced levels of 9 genes found to be activated >2-fold by nutlin-3a in early passage WI-38 cells.  Nutlin treatment produced comparable amount of p53 protein in early passage and senescent cells but it induced different mRNA levels in the two cell populations (Figure 3A, 3B).  Eight out of nine genes were induced to lower levels in the senescent cells, and this status remain unchanged after normalization for total gene expression (Figure 3B, 3C), suggesting an overall decrease in p53 transcriptional activity.  Similar observations were made with nutlin-induced expression of p53 regulated microRNA genes. All 4 microRNA genes were induced to lower levels in senescence (Figure 3D, 3E).  Since nutlin does not require upstream signaling and produced practically equivalent amounts of p53 in both cell populations, these results lead to the conclusion that the ability of p53 to activate its transcription targets is compromised in senescent cells.

To assess the apoptotic function of p53 in senescent WI-38 cells we used the DNA-damaging drug doxorubicin.  It has been shown previously that nutlin-3a effectively induces apoptosis in cancer cells despite the lack of phosphorylation on key serine residues [25,26].  However, it is only growth suppressive in normal fibroblasts [22].  Doxorubicin treatment induced moderate apoptosis in early passage cells but only slight increase over the controls in senescent WI-38 cells (Figure 4A).  The observed decline in apoptotic activity is in agreement with previous reports [17,20] and likely reflects the inefficient stabilization of p53 in senescent cells (Figure 4B).   The fact that nutlin induced comparable p53 protein level in early passage and senescent cells (Figure 3A) confirms previously suggested mechanism that the decline in transcriptional activity in response to DNA damage is due to defective upstream signaling leading to p53 stabilization.  Consistent with the decrease in p53 levels, induction of p53 target genes by doxorubicin was reduced in senescent cells (Figure 4C, D), especially in apoptosis related genes, which might contribute to apoptosis resistance.   We have shown previously that nutlin enhances p53 stabilization by doxorubicin [33].  The combination of nutlin and doxorubicin in senescent cells raised p53 protein and this increase in p53 levels restored the loss in transcriptional (Figure 4G, 4H) and apoptotic activity (Figure 4F) in senescent WI-38 cells, pointing out to inefficient p53 stabilization as a major contributor to the decline in apoptotic activity in response to DNA damage. The reasons for still incomplete restoration of apoptotic activity are not clear but one can speculate that other p53-independent events induced by the genotoxic drug and possibly altered during senescence are also contributing to the apoptotic response.

Our analysis of p53 pathway functionality in senescent WI-38 fibroblasts showed overall decline in transcriptional and apoptotic activity.  This decline may result from changes in the upstream signaling leading to inefficient p53 stabilization and compromised transcriptional activation of target genes but also attenuated downstream apoptotic signaling.  The molecular mechanisms behind these changes are currently obscure and warrant further investigation using specific p53 probes such as nutlin.   While the fundamental reasons for the decline in p53 activity are unclear, one can speculate that p53 pro-apoptotic function is redundant in senescent cells which have already lost their ability to proliferate and hence to become cancerous.

## Materials and methods

Cell culture and drug treatment. WI-38 human diploid fibroblast was purchased from ATCC and cultured in minimal essential medium (MEM) supplemented with 10% fetal bovine serum and 1 mM sodium pyruvate. Cells were kept in exponential growth by passaging twice a week.  Cells below passage 10 were designated "early passage cells".  Doxorubicin was purchased from Sigma and Nutlin-3a was synthesized at Hoffmann-La Roche Inc., Nutley, NJ.  Both agents were dissolved in DMSO as 10 mM stock solution and kept frozen in aliquots.

Western blotting. Western blottings was performed as previously described [33].  Primary antibodies used are as follow: p53 (sc-263) and MDM2 (sc-965) were from Santa Cruz Biotechnology, p21 (OP64) was from Calbiochem, p53-Ser15 was from Cell Signaling Technology.

Quantitative real-time PCR. To quantify mRNA expression level, total RNA was isolated from cells using RNeasy kit (Qiagen). 2 μg of RNA was converted to cDNA using the TaqMan RT kit (Applied Biosystems). Taqman quantitative real-time PCR analysis was performed using ABI PRISM 7900HT detection system from Applied Biosystems. The Q-PCR expression assay for p53 target genes (APAF1, BAX,  BBC3, BTG2, CDKN1A, FAS, FDXR, GDF15, IL8, MDM2, NUPR1, PERP, PMAIP1,, SERPINE1, TNFRSF10B,  TP53I3, TP53INP1, ZMAT3), and two controls (18S rRNA, GAPDH) as well as E2F1 were built into TaqMan® Custom Array (Applied Biosystems).  To quantify microRNA expression, total RNA was isolated using the TRIzol solution (Invitrogen) following manufacturer's instruction. RNA was converted to cDNA using the TaqMan microRNA Transcription Kit (Applied Biosystems), and real-time PCR analysis was performed using TaqMan microRNA assays (Applied Biosystems). Expression levels were normalized to an internal control, RNU48.

Cell cycle analysis . BrdU (20 μM, Sigma) was added to the cells 1 hour before cell collection.  Cells were fixed in 70% ethanol at -20˚C for 1h, permeablilized with 2N HCl and 0.5% Triton X100 for 30 minutes, and neutralized with 0.1 M sodium tetraborate. Cells were then stained with anti-BrdU FITC-conjugated antibody and propidium iodide (Becton Dickinson) for cell cycle analysis using FACScalibur flowcytometer (Becton Dickinson, Franklin Lakes, NJ).

Apoptosis and senescence assays. Cells were seeded in 6-well plates (1х10^5^) Apoptosis was determined with Guava NexinTM Kit using the Guava Personal Cell Analyzer (Guava Technologies, Hayward, CA). SA-β-Gal activity was measured with the Senescent Cell Staining kit (Sigma, St. Louis, MO) according to manufacturer's instructions. Stained cells were visualized with the Nikon Eclipse TE 2000U microscope and images were taken by the Nikon Digital Camera DXM 1200F.  For SAHF detection, cells were cultured in chamber slides, fixed with 4 % formal-dehyde, permeabilized with 0.1% Triton X-100, and blocked with 1% BSA. Primary antibody anti-HP1γ (1:200) and secondary antibody anti-rabbit IgG(H+L) F(ab')2 fragment (DyLightTM 488 conjugate) were from Cell Signaling Technology. Prolong® Gold Antifade Reagent containing DAPI (Invitrogen) was applied to stain DNA.
